# Prevalence and factors associated with psychological distress among patients on warfarin at the Uganda Heart Institute, Mulago Hospital

**DOI:** 10.1186/s12888-022-03998-w

**Published:** 2022-05-20

**Authors:** Molly Naisanga, Christine SekaggyaWiltshire, Wilson Winstons Muhwezi, Joseph Musaazi, Dickens Akena

**Affiliations:** 1 Gulu University, Gulu, Uganda; 2 Infectious Disease Institute, Kampala, Uganda; 3 Makerere University, Kampala, Uganda

**Keywords:** Warfarin, Psychological distress, Cardiovascular disease, Anticoagulants

## Abstract

**Introduction:**

Cardiovascular diseases (CVD) are the leading cause of mortality worldwide and are significantly associated with multiple comorbid disorders including mental disorders such as psychological distress (PD). At increased risk of PD are CVD patient sub-categories that not only require chronic therapy but also need follow up with continuous blood tests and dose adjustments (like the patients on warfarin). However, not much has been done to ascertain the burden of PD among patients on warfarin in Uganda.

**Objective:**

To determine the prevalence and factors associated with PD among patients on anticoagulation with warfarin at the Uganda Heart Institute (UHI).

**Methods:**

In this analytical cross-sectional study, 197 participants were sampled from adults on warfarin attending the Uganda Heart Institute (UHI) out patient clinic. The Self Reporting Questionnaire (SRQ-20), a tool with a total maximum score of 20 and cutoff for PD at ≥6 was used to determine the presence of PD among participants, and a socio-demographic questionnaire to document the socio-demographic characteristics of the subjects. Additional questions including the underlying CVD diagnosis, medications used (besides warfarin) and presence of chronic illnesess were also assessed. Bi-variable and multi-variabe logistic regression analysis techniques were used to examine the associations between the dependent and independent variables.

**Results:**

The prevalence of PD was 32%. The unemployed participants were 4.5 times more likely to have PD (adjusted odds ratio [aOR]4.56, 95% confidence interval [CI]: 1.12–18.62, *p* = 0.04). Participants who had experienced social stressors were more likely to have PD (aOR: 11.38, CI: 3.60–36.04, *p* < 0.01). Other factors associated with a higher likelihood of having PD included: presence of other chronic comorbidities (aOR: 3.69, CI: 1.24–11.02, *p* = 0.02) and concomitant use of loop diuretics (aOR: 4.13, CI: 1.67–10.19,*p* < 0.01). A shorter length of time on warfarin (7–24 months) lowered the likelihood of PD (aOR: 0.23, CI: 0.07–0.74, *p* = 0.01).

**Conclusion:**

The prevalence of PD was high among patients on warfarin in this low income setting and there is a need to characterize the specific psychiatric disorders in patients with CVD. Interventions that address the high burden of PD are urgently needed in this setting.

**Supplementary Information:**

The online version contains supplementary material available at 10.1186/s12888-022-03998-w.

## Background

A number of global burden of disease (GBD) surveys have shown cardio vascular diseases (CVD) to be the leading cause of mortality and morbidity worldwide, accounting for one third of all deaths as of 2015 [1], (https://www.who.int/news-room/fact-sheets/detail/cardiovascular-diseases-(cvds). A number of individuals with CVD may develop thrombo-embolic states including deep venous thrombosis, pulmonary embolism, ischemic stroke and ischemic heart disease (IHD). In low and middle income countries (LMIC), about 1.1 per 1000 individuals are affected by thrombo-embolic ailments [2]. Patients with these conditions are managed for extended periods of time on anticoagulants like warfarin [3].

A number of individuals with CVD will suffer from a mental illness during the course of their illness [4]. Similarly, patients with mental illness have an increased risk for CVD [5] and an increased prevalence of CVD [6]. A number of mechanisms have been identified to directly or indirectly lead to cardiovascular disease in this population. These may include: (i) derangements in the body cardiovascular physiology during the course of some mental illnesses, such as increased cardiac reactivity (increased heart rate and blood pressure), reduced blood flow to the heart, and heightened levels of cortisol; (ii) cardiometabolically undesirable side effects (like dyslipidemia, glucose intolerance, weight gain,among others) of many medications used to treat mental illness [7, 8]; (iii) unhealthy lifestyles related to diet, smoking, physical activity, among others that are highly prevalent among patients with mental illnesses [9, 10]. These mechanisms play a role in the occurance of CVD among patients with mental illness.

In another category of individuals, mental illnesses arise during the course or post the cardiovascular disease [4]. Most common in this category is psychological distress (PD) often associated with anxiety and depression [11, 12]. PD as an entity has been defined as a state of emotional suffering characterized by symptoms of depression (excessive and persistent sadness, anhedonia, hopelessness, among others) and anxiety (restlessness, feeling tense, sleep disturbance, among others) [13]. The likelihood of occurance of PD has also been associated with a number of factors including gender, negative stressful life events like major life events, trauma and other stressors [14–16].

The association between CVD and PD has been shown to be bi-directional in nature [9]. PD may significantly contribute to the etiology of the CVD by inducing a hypercoagulable state through platelet abnormalities, hemoconcentration and increased catecholamines concentration which results into a heightened sympathetic arousal [17–19]. PD may also result from the CVD and the factors associated with it including uncertainty about the disease prognosis and reliance on life-long treatment [20]. At increased risk of developing PD is the sub-category of individuals on warfarin that not only require chronic therapy (for the primary CVD diagnosis), but have increased use of medical care services in the form of follow up with continuous blood tests and dose adjustments so as to achieve target ranges like the international normalized ratio (INR). Notably, increased medical care use has been associated with PD [12].

Warfarin is a coumarin administered orally for the prevention of thromboembolic events. The presence of PD among individuals on warfarin is likely to be associated with poor adherence and increased mortality as has been the case among other populations on chronic medication like patients on Highly Active Anti-Retroviral Therapy (HAART) [21]. However, little has been done in sub-Saharan Africa (SSA) to document the burden of PD among patients with CVD on warfarin. For this study, the prevalence of PD and factors associated with it was examined in persons with CVD taking warfarin at the Uganda Heart Institute (UHI), a low resourced SSA setting where disease burden is high and the prevalence of CVD is documented to be on the increase. This is among the preliminary studies in sub-Sahara Africa of its kind and these findings will be used to improve clinical practice.

## Methods

The study was an analytical cross-sectional study.

### Study site

Study participants were recruited from the Uganda Heart Institute (UHI), a private specialized unit within Mulago National Referral Hospital that diagnoses, treats, and manages cardiac diseases, including birth abnormalities, venous thrombo embolism and coronary artery disease. The UHI is the largest and most advanced center for CVD care in Uganda and receives referrals from allover the country. As part of outpatient services, about 400 patients on warfarin are attended to.

### Study population

This study included adults on anticoagulation with warfarin at the UHI outpatient clinic who consented to participate in the study from 06/2019 to 09/2019. Patients who were unable to volunteer information during the time of the study due to severe illness with inability to sustain an interview or had an emergency life threatening situation, for which urgent medical attention was needed were excluded from the study. We consecutively sampled 197 participants. This was calculated using the Leslie Kish formula (with a standard error at 5%, proportion of patients at 50% and a standard normal deviation of 1.96). We also corrected for the accessible population (there are about 400 patients on warfarin at the UHI) [22] using the Conchrane finite population correction.

### Study measures

#### The self-reporting Questionaire (SRQ-20)

The primary outcome was PD. All enrolled participants were assessed using the SRQ-20, and significant PD was defined as a score of 6 and above. The SRQ-20 was developed by the World Health Organization (WHO) particularly for use in LMIC countries to detect non -specific PD [23] . The SRQ-20 consists of 20 items designed to identify common mental disorders. It focuses on the individual’s feelings over the past 30 days. It explores variables like somatic symptoms of mental illness (headache, sleep, appetite, tremulousness among others) and mood symptoms (like unhappiness, loss of interest in previously enjoyed activities, feeling tense, among others). A score of 1 indicates that the symptom was present; a score of 0 indicates the symptom was absent, with a maximum possible score of 20. The SRQ-20 has been translated to the local language- Luganda, in Uganda (with adaptation and validation); a score of 6 and above has yielded a good sensitivity (75–84%) and a good specificity (74–93%) in detecting psychiatric comorbidity. These psychometric properties have been established in the primary health care setting and in the context of HIV care among patients on HAART [24, 25] . The locally adapted version was used in this study and PD was defined as an SRQ-20 score of 6 and above.

Socio-demographic characteristics were assesed using a structured questionnaire and included age, gender, education level, marital status, occupation and religion. Factors relating to underlying CVD illness like underlying CVD diagnosis, duration on warfarin (in months), comorbid illnesses among others were assessed by reviewing the clinical records of the patients. Other personal and environmental factors like major life events (loss of a loved one, retrenchment or divorce), trauma (sexual abuse, violence- thuggery related or domestic) among others were assessed by use of a structured questionnaire. Participants were asked if they had experienced any of these events at any one time during the 6 months preceding the time of interview.

### Statistical analysis

To determine the prevalence of PD among patients on warfarin attending the UHI outpatient clinic, the number of patients with PD out of the total number of study participants was expressed as a percentage. Bivariable analysis of factors associated with PD was done, odds ratios and their 95% confidence intervals were obtained.

A Multivariable logistic regression model was built to examine the association between PD and its predictors while controlling for gender and age. Variables with a *p*-value of < 0.2 at bivariable level were added to the model using stepwise elimination to obtain a model of best fit. Variables were tested for multicollinearity before being included in the model. Goodness of fit tests were conducted at 5% level of significance. Variables that achieved a *p* < 0.05 were considered significant. The results are displayed in text, tables and figures.

Further still, we used the variance inflation factor (VIF) to check for multicollinearity using cut-off of VIF > 5. We used Hosmer-Lemishow to check for goodness of fit in the final model.

## Results

### Socio-demographic characteristics of the study participants

Data was collected from 197 participants. Most of the participants 132 (67%) were female. The median age was 37 years (IQR: 27–50) and about one fifth of the participants [32 (16.2%)] were 55 years and above. There was an equal distribution of participants between elementary/informal education and secondary level with 64 participants (32.5%) in each category. Majority of the participants [117 (59%)] were married. The socio-demographic characteristics are detailed in Table 1.

**Table 1:** Socio-demographic characteristics of participants

Variables	Frequency (***N*** = 197)	Percentage
**Gender**		
Male	65	33.0
Female	132	67.0
**Age**		
18–34	87	44.2
35–54	78	39.6
55+	32	16.2
**Education level**		
Informal/elementary	64	32.5
Secondary	64	32.5
Post secondary	69	35.0
**Marital status**		
Single	80	40.6
Married	117	59.4
Employment*		
Employed full time	46	24.0
Self employed	88	45.0
Un employed	41	21.0
Student	20	10.0
Religion*		
Christian	140	72.0
Muslim	17	9.0
Others	38	19.0

*variables with missing data

### Clinical characteristics of the participants

Table 2, the indication for use of warfarin in 98 (50.8%) of the study participants was a diagnosis of rheumatic heart disease (RHD). The majority of the participants 161 (82%) had no other comorbidity besides the one for which warfarin had been prescribed. Approximately one third of the participants had a positive history of cardiac related surgery. Several patients had also experienced other life stressors in the previous 6 months, most commonly occupational stressors (work overload, and work related deadlines, lack of employment) in ninety eight (49.6%) of the participants and least commonly trauma (sexual abuse and robbery related or domestic violence) in 17 (8.6%) of the participants.

**Table 2:** Clinical factors and life stressors

Variables	Frequency	Proportion (%)
**Underlying CVS diagnosis**		
Rheumatic heart disease	98	50.8
Atrial fibrillation	53	27.5
Thrombo-embolism	30	15.6
Others	12	6.1
**Length of time on Warfarin**		
≤ 6	39	20.9
> 6–24	57	30.5
25–60	56	30.0
> 60	35	18.7
**Other comorbidities**		
Present	36	18.3
None	161	81.7
**History of cardiac surgery**		
Yes	58	30.0
No	138	70.0
**Length from time of surgery in months (*N*** **= 58)**		
< 1 year	12	21.1
1 to < 3 years	22	38.6
3 to 5 years	10	17.5
> 5 years	13	22.8
Other chronic medications besides Warfarin*		
Loop diuretic	108	63.5
Penicillin	93	54.7
Beta blocker	86	50.8
Cardiac glycoside	64	37.7
Others	15	64.5
Life stressors*		
Major life events	47	23.9
Trauma	17	8.6
Economic stressors	38	19.3
Social stressors	36	18.3
Occupational stressors	98	49.8

One participant could have more than one medication or stressor*

### Factors associated with PD

At bivariable analysis; increasing age, being self employed/unemployed were associated with increased likelihood of PD. Increasing education attainment decreased the likelihood of PD. The details of the bivariable analysis of the socio-demographic characteristics are given in Table 3. Among clinical factors, at bivariable analysis; compared to having rheumatic heart disease, having atrial fibrillation increased the likelihood of PD. Other factors which increased the likelihood of PD at bivariable analysis were; having a comorbidity, use of loop diuretics and experience of a life stressor. The details of the bivariable analysis of the clinical characteristics and life stressors (major life events, traumatic events, economic stressors, social stressors and occupational stressors) are given in Tables 4 and 5 respectively. At multivariable analysis, the unemployed (adjusted odds ratio [aOR]: 4.56, 95% confidence interval [CI]:1.12–18.62, *p* = 0.04) and students (aOR: 8.02, CI: 1.51–42.69, *p* = 0.02) had an increased likelihood of being psychologically distressed. A shorter length of time on warfarin decreased the likelihood of PD (aOR: 0.03, CI: 0.07–0.74, *p* = 0.04). Participants with other chronic comorbidities were almost 4 times more likely to be psychologically distressed (aOR: 3.69, CI: 1.24–11.02, *p* = 0.02), as well as persons who were concomitantly on loop diuretics (aOR: 4.13, CI: 1.67–10.19, *p* < 0.01). Participants who had experienced social stressors were 11 times more likely to have PD (aOR:11.38,CI: 3.60–36.04, *p* < 0.01). The details of the multivariable analysis for the factors associated with PD are given in Table 6. The internal consistency of the SRQ-20 in this study was 0.85.

**Table 3:** Bivariable socio-demographics analysis

	PD present ***n*** = 63 (32%)	PD absent ***n*** = 134 (68%)	OR (95% CI)	***P*** value
**Gender**				
Male	24 (36.9)	41 (63.1)	1	
Female	39 (29.6)	93 (70.5)	0.72 (0.38–1.34)	0.38
**Age**				
18–34	18 (20.7)	69 (79.3)	1	
35–54	25 (32.1)	53 (68)	1.81 (0.89–3.65)	0.10
55+	20 (62.5)	12 (37.5)	6.39 (2.64–15.5)	< 0.01
**Education**				
Informal/primary	28 (43.8)	36 (56.25)	1	
Secondary	21 (32.8)	43 (67.2)	0.63 (0.31–1.29)	0.20
Post secondary	14 (20.3)	55 (79.7)	0.33 (0.15–0.70)	< 0.01
**Marital status**				
Single	27 (33.8)	53 (66.3)	1	
Married	36 (30.8)	81 (69.2)	0.87 (048–1.60)	0.66
**Employment**				
Employed full time	7 (15.2)	39 (84.8)	1	
Self employed	32 (36.4)	53 (63.6)	3.18 (1.28–7.94)	0.01
Un employed	17 (41.5)	24 (58.5)	3.95 (1.43–10.91)	0.01
Student	6 (30.0)	14 (70.0)	2.39 (0.68–8.33)	0.17
**Religion**				
Christian	48 (34.3)	92 (65.7)	1	
Muslim	6 (35.3)	11 (64.7)	1.05 (0.36–3.00)	0.93
Others	9 (23.7)	29 (76.3)	0.59 (0.37–0.74)	0.22

**Table 4:** Association of PD with participants’ clinical characteristics

	PD present ***n*** = 63 (32%)	PD absent ***n*** = 134 (68%)	OR (95% CI)	***P*** value
**Underlying CVD diagnosis**				
Rheumatic heart disease	22 (22.5)	76 (77.6)	1	
Atrial fibrillation	22 (41.5)	31 (58.5)	2.45 (1.19–5.06)	0.02
Thrombo-embolism	9 (30.0)	21 (70.0)	1.48 (0.59–3.69)	0.40
**Length of time on Warfarin**				
≤ 6 months	17 (43.6)	22 (56.4)	1	
7–24 months	12 (21.1)	45 (78.9)	0.35 (0.14–0.85)	0.02
25–60	15 (26.8)	41 (73.2)	0.47 (0.20–1.13)	0.09
> 60	13 (37.1)	22 (62.9)	0.76 (0.30–1.94)	0.57
**Other comorbidities**				
No comorbidity	44 (27.3)	117 (72.7)	1	
Any comorbidity	19 (52.8)	17 (47.2)	2.97 (1.42–6.23)	< 0.01
**History of cardiac surgey**				
No	40 (29.0)	98 (71.0)	1	
Yes	22 (37.9)	36 (62.1)	1.50 (0.79–2.85)	0.22
**Length from time of surgery**				
< 1 year	4 (33.3)	8 (66.7)	1	
1 to < 3 years	9 (40.9)	13 (59.1)	1.38 (0.32–6.03)	0.67
3 to 5 years	4 (40.0)	6 (60.0)	1.33 (0.23–7.63)	0.75
> 5 years	5 (38.5)	8 (61.5)	1.25 (0.24–6.44)	0.79
**Chronic medication beside Warfarin**				
**Loop diuretic**				
No	20 (21.7)	72 (78.3)	1	
Yes	43 (41.0)	62 (59.0)	2.50 (1.33–4.69)	< 0.01
**Penicillin**				
No	71 (63.4)	41 (36.6)		
Yes	22 (25.9)	63 (74.1)	0.60 (0.33–1.12)	0.11
**Beta blocker**				
No	83 (70.3)	35 (29.7)	1	
Yes	28 (35.4)	51 (64.6)	1.30 (0.71–2.39)	0.39
**Cardiac glycoside**				
No	41 (29.7)	97 (70.3)	1	
Yes	22 (37.3)	37 (62.7)	1.40 (0.74–2.67)	0.30

**Table 5:** Association of PD with life stressors

	PD present ***n*** = 63 (32%)	PD absent ***n*** = 134 (68%)	OR	***P*** value
**Life stressors**				
Major life events				
No	35 (23.3)	115 (76.7)	1	
Yes	28 (59.6)	19 (40.4)	4.84 (2.42–9.70)	< 0.01
Trauma				
No	54 (30.0)	126 (70.0)	1	
Yes	9 (52.9)	8 (47.1)	2.63 (0.96–7.17)	0.06
Economic stressors				
No	43 (27.0)	116 (73.0)	1	
Yes	20 (52.6)	18 (47.4)	3.00 (1.45–1.6.20)	< 0.01
Social stressors				
No	40 (24.8)	121 (75.2)	1	
Yes	23 (63.9)	13 (36.1)	5.35 (2.48–11.54)	< 0.01
Occupational stressors				
No	37 (37.4)	62 (62.6)	1	
Yes	26 (26.5)	72 (73.5)	0.61 (0.33–1.11)	0.10

**Table 6:** Multivariable logistic regression of factors associated with PD

	AOR(95% CI)	***P***-value
**Gender**		
Male	1	
Female	0.76 (0.31–1.91)	0.56
**Age**		
18–34	1	
35–54	0.9 (0.33–2.68)	0.90
55+	1.73 (0.46–6.44)	0.42
**Education level**		
Informal /primary	1	
Secondary	0.60 (0.22–1.66)	0.32
Post secondary	0.42 (0.14–1.29)	0.13
**Employment**		
Employed full time	1	
Self employed	1.86 (0.56–6.19)	0.31
Un employed	4.56 (1.12–18.62)	**0.04**
Student	8.02 (1.51–42.69)	**0.02**
**Length of time on warfarin**		
≤ 6 months	1	
7–24 months	0.23 (0.07–0.74)	**0.01**
25–60 months	0.34 (0.11–1.06)	0.06
> 60 months	1.14 (0.35–3.72)	0.83
**Other comorbidities**		
No	1	
Yes	3.69 (1.24–11.02)	**0.02**
**Loop diuretic**		
No	1	
Yes	4.13 (1.67–10.19)	**< 0.01**
**Social stressors**		
No	1	
Yes	11.38 (3.60–36.04)	**< 0.01**

### Multicollinearity check in final model

The mean variance inflation factor (VIF) in the final model was 2.02, indicating no multicollinearity among factors retained in the final model. *P*-value for goodness of fit in the final model was 0.8566, indicating no evidence to reject null hypothesis and that the model had a good fit.

## Discussion

We set out to determine the prevalence and factors associated with PD among patients on warfarin at a private outpatient clinic in urban Uganda. This is one of the first studies of its kind in Uganda and SSA. We found a high prevalence of PD at 32.0%. These results are similar to a study done in Melbourne in a tertiary centre just like UHI where the prevalence of PD was found at 35% [26]. However, the study in Melbourne was restricted to patients with atrial fibrillation - the most studied population often on warfarin (27.5% of participants in this study) and a different tool (Hospital Anxiety and Depression Scale) was used to measure psychological distress. In this study we included patients with several other diagnoses provided they were on warfarin and still got similar results indicating that PD is highly prevalent in this population.

A study in another population on chronic medication (HAART) in Uganda found a similar prevalence of PD at 30.3% [21]. This prevalence was much higher than that found among patients on HAART in Ethiopia [27] where a higher cutoff score of defining PD on the SRQ-20 was used and prevalence of PD found at 7.8%. The investigators in Ethiopia used a cutoff score of 11 and above which is much higher than the score of 6 and above which was used in this study. This could explain the lower prevalence of PD found among patients on HAART in Ethiopia.

Being unemployed was found to increase the chances of having PD. Studies among other cardiovascular disease populations like those with myocardial infarction [28] have corroborated these findings and so have studies among patients on HAART [29]. The association between PD and unemployment may be explained by difficulties in accessing health care services, a reflection of the financial strain commonly present among the unemployed [30]. Accessing health care services is even made more difficult by the inadequate health care services availed to patients with CVD in Uganda [22, 31] and often times patients have to incur out of pocket expenses to meet their healthcare needs. Similar circumstances (difficulties in accessing health care services) may be responsible for the association between PD and being a student.

Other studies exploring the relationship between PD and unemployment have found no association between PD and unemployment [32]. Important to note, these were conducted among populations where access to health care services is not an immediate concern as may occur in the general population which was the population studied by these investigators .

Presence of social stressors (strained relationships and family changes) was positively associated with PD similar to studies in other populations on chronic medication like patients on HAART [29]. A relationship between social stressors and psychological well being has been discussed in detail [33]; it has been argued that proper functioning of social relationships provides informational, emotional and instrumental resources that are very key in the context of cardiovascular diseases including when on warfarin medication [20]. Absence of these relationships puts one at increased risk of PD through several biopsychosocial mechanisms (like maladaptive coping and dysregulation of the hypothalamic-pituitary-adrenal axis functioning) that are key in determining occurance of PD disorders [34].

Presence of any other chronic comorbidity (a non-cardiovascular disease) was positively associated with PD in this study. The presence of comorbidities has been documented in persons with CVD in a number of studies [35, 36]. It’s association with PD is similar to previous findings in other populations on chronic medication like patients on treatment for tuberculosis. This may be attributed to the labelling effect that comes with having these illnesses, perceived deterioration in one’s state of health, increased healthcare utilization that comes with the comorbidities, not with standing the fact that having more than one co-occuring ailment is an additional psychological stress to an individual [37]. Indeed patients with comorbidities will be on more medication as compared to those without comorbidity.

Use of several medications is of concern because particular non-psychotropic medications; antihypertensives, antiobesity drugs, interferons, corticosteroids [38, 39] have been noted to be associated with PD disorders (depression and anxiety disorders). Of all implicated medications, most relevant to this study are the loop diuretics class of antihypertensives which were significantly associated with PD. Previous literature indicating an association between PD and loop diuretics is scanty hence the need for further studies to explore this relationship.

A shorter length of use of warfarin was found to be poorly associated with PD. There were no immediate studies to compare these findings with but this could be explained by the possibility that a shorter length of time on warfarin could be a marker of less severe disease or better prognosis. Unlike previous studies in which female gender was associated with PD [16, 28], this association was not established in this study.

### Limitations of the study

The study was cross-sectional in design, therefore it could not determine the causal relationship between PD and its associated factors. Further longitudinal studies are required to examine the causal relationship between PD and the associated factors. The Self Reporting Questionnaire has not been validated in this particular population in this setting though it has been used among patients on HAART here in Uganda a population on chronic medication just like the patients on warfarin investigated in this study.

## Conclusion

There is a high prevalence of psychological distress among patients receiving warfarin. The likelihood of PD in these patients is increased among those who are unemployed, students, those with several comorbidities, patients on loop diuretics and those who have experienced social stressors. Considering these findings, screening for PD in tertiary health care facilities among patients receiving warfarin should be encouraged. Special attention should be given to those where the likelihood of PD is increased. Further still, efforts should be made to provide the necessary support for these patients to mitigate the possibility of psychological distress..

## Supplementary Information

**Additional file 1.**

**Additional file 2.**

**Additional file 3.**

**Additional file 4.**

## Data Availability

All data generated or analysed during this study are included in this published article as supplementary information files.

## Abbreviations

CVD: Cardiovascular diseases
DVT: Deep Venous Thrombosis
GBD: Global Burden of Disease
PD: Psychological Distress
PE: Pulmonary Embolism
RHD: Rheumatic Heart Disease
INR: International Normalized Ratio
LMIC: Low and Middle Income Countries
SSA: Sub-Sahara Africa
SOMREC: School of Medicine Research and Ethics Committee
UHI: Uganda Heart Institute
VTE: Venous Thrombo Embolism
WarPATH: Warfarin Anticoagulation in Patients in Sub-Saharan Africa
