# Supplementary material for: Prevalence and factors associated with psychological distress among patients on warfarin at the Uganda Heart Institute, Mulago Hospital
Source: BMC Psychiatry. 2022 May 20;22:349. doi: 10.1186/s12888-022-03998-w (PMC9123720; doi:10.1186/s12888-022-03998-w)
Supplement: Supplementary file 3 — Additional file 3. [file 12888_2022_3998_MOESM3_ESM.docx]

**UNDERLYING CARDIOVASCULAR DIAGNOSIS**

Afib 2

AVR 11

Dilated cardiomyopathy 5

DVT 3

Heart failures 10

IHD 7

Mitral valves regurgitation 9

PHT transition AV canal defect 8

Pulmonary embolism 6

RHD 1

Right ventricular endocardial fibrosis 4

Unclear 0

**OTHER COMMORBIDITIES**

Cancer 4

DM 6

Dyslipidemia 5

Erectile dysfxn 2

HIV 3

HTN 1

PYD 7

Asthma 8

OTHER CHRONIC MEDICATIONS BESIDES WARFARIN

| DRUG | CLASS | NUMBER |
| --- | --- | --- |
|  |  |  |
| Amiodarone | Class III anticrhythim drug | 17 |
| Amlodipine (casomex) | Ca channel blocker | 15 |
| Antacorist | Endothelin | 11 |
| Anticancer | Anticancer | 10 |
| Arbitel | Arbitel | 14 |
| Aspirin | Aspirin | 8 |
| Atenolol | Betal blocker | 2 |
| Benzathin pencillin | Pencillin | 1 |
| Biso prolol | Betal blocker | 2 |
| Bosertan | Endothelin | 11 |
| Cave dilol | Betal blocker | 2 |
| Concor | Betal blocker | 2 |
| Digoxin | Cardiac glycoside | 5 |
|  |  |  |
| Enalapril | ACE | 6 |
| Enoxaparin aka dexare | Low molecular hepan | 8 |
| Haart | Haart | 13 |
| Lasix | Loop diuretic | 4 |
| Losartan | ARB | 3 |
| melformn | Oral anti DM | 16 |
| Metalazore Y/N | Thiazide like diuretic | 9 |
| Prednisolse | Steroids | 18 |
| Ramipril | ACE | 6 |
| Sildenatil | PDE | 12 |
| spironolactue | Potassium spang diuretic | 7 |
| telmisartan | ARB | 3 |
| zelmisartan | ARB | 3 |
| Pen V | Pencillin | 1 |
| verapamil | ca channel blocker | 15 |
| Others | State-H | 19 |
